# Implications for balance in 11- and 12-year-old children in northern Spain during SARS-CoV-2 lockdown

**DOI:** 10.3389/fpsyg.2022.1009299

**Published:** 2022-09-21

**Authors:** Oliver Ramos-Álvarez, Víctor Arufe-Giráldez, Alberto Sanmiguel-Rodríguez, Rubén Navarro-Patón

**Affiliations:** ^1^Education Faculty, University of A Coruña, A Coruña, Spain; ^2^Departamento de Educación, Área de Educación Física y Deportiva, Universidad de Cantabria, Santander, Spain; ^3^Research Unit of School Sports, Physical Education and Psychomotricity (UNIDEF), Specific Didactics Department, Research and Diagnostic Methods in Education, Education Faculty, University of A Coruña, A Coruña, Spain; ^4^Faculty of Language and Education, University of Camilo José Cela, Madrid, Spain; ^5^Facultad de Formación del Profesorado, Universidade de Santiago de Compostela, Lugo, Spain

**Keywords:** SARS-CoV-2, physical activity, children, MABC-2, balance

## Abstract

**Introduction:**

The home lockdown due to the appearance of SARS-CoV-2 in Spanish society led to changes in certain habits in children and adolescents. These habits were related to the practice of physical activity and the implications of higher rates of sedentary activities. This lockdown lasted from March to June 2020. The aim of this study was to determine the implication that lockdown in Spain due to the SARS-CoV-2 virus outbreak had on balance in 11–12 year-old schoolchildren.

**Materials and methods:**

In total, 50 Spanish children aged 11–12 years (*M* = 11.40, SD = 0.50) participated, 33 (66%) boys and 17 (34%) girls. The Movement Assessment Battery for Children 2 (Movement ABC-2) and an *ad hoc* questionnaire for sociodemographic data and other relevant information were used for the three data collections.

**Results:**

There are significant differences (*p* < 0.05) in the results for balance variables measured by static balance tests on supports in the total sample, in boys and girls. There are also significant differences in the total sample as well as in the boys in the heel-toe backward walking test. In addition, there are significant differences in mean, scalar and percentile dimension scores for balance between before and after lockdown in both boys and girls. No significant differences were found in the total sample or by gender in measurements related to the zigzag jumping test with dominant as well as non-dominant leg in girls (*p* = 0.317).

**Conclusion:**

As a consequence of SARS-CoV-2 confinement, there was a worsening of balance values in children aged 11–12 years.

## Introduction

During the health crisis caused by COVID-19, many countries around the world opted for unusual measures to contain the virus that was spreading uncontrollably. Spain, like other neighbouring countries such as Italy, France and Portugal, opted to impose social measures of home lockdown to eradicate the transmission of the virus among the population. Spain by Royal Decree 463/2020 of 14 March declaring a state of alarm for the management of the health crisis caused by COVID-19 and restricting the movement of the entire Spanish population, except for essential workers (Agencia Estatal Boletín Oficial del Estado, 2020) began house confinement. These restrictions were in force for 98 days between 15 March and 21 June 2020. Educational establishments and sports schools were closed without a specific date for their reopening. During the course of this period of home confinement, the population in general and children and adolescents in particular modified their habits in relation to daily physical activity (PA), screen time, and eating habits (Arufe-Giráldez et al., 2020; Reina Prieto, 2020; Ramos Álvarez et al., 2021a). These changes resulted in changes in children’s anthropometric averages, physical condition and psychological and emotional states (Quero et al., 2021; Ramos Álvarez et al., 2021a,b, 2022).

In this context, the development of motor competence is affected in children and young people. Motor competence is the implementation of a motor skill as a response in a given context (Fort-Vanmeerhaeghe et al., 2017). Balance is an important part of motor competence as an essential element of human movement as well as for body control. The good development of balance is fundamental for the correct development of general dynamic coordination and for the autonomy of the different body segments (Ruiz-Pérez, 2004). The development of motor competence is associated with the period of human growth and maturation, but there are certain life events that can create a worsening of human motor competence, such as ageing itself or the absence of physical activity (Fort-Vanmeerhaeghe et al., 2017).

Following the explanatory models of motor development of authors such as Gallahue, addressed in the work of Ruiz Perez (Ruiz-Pérez, 2004; Gallahue and Ozmun, 2006; Rigal, 2006; Ruiz-Pérez et al., 2014), or Clark and Metcalf (2002), the process of motor development is composed of different factors. These factors are individual factors (e.g., biological), task factors (e.g., physical characteristics or cognitive requirements). And finally, contextual factors, in which we find socio-cultural factors and which to a large extent can condition this motor development and as a consequence of the balance values. Through the active participation of the child, it promotes their motor development and therefore their balance, thus producing this social, affective, educational and ecological stimulation. In addition, motor experiences become essential to prevent possible deficiencies or impairments in motor development (Gardner, 1976; Young and Fujimoto Gómez, 2004). For example, disadvantaged socio-economic situations, living in small dwellings or without sufficient space, are considered limiting factors for the correct motor and physical development of the child development (Ruopp et al., 1979).

But if we look even more closely at the factors that influence the stability of balance, another series of factors can be identified: psychological and environmental factors, factors related to physical condition, physiological factors, and mechanical factors (García-López and Rodríguez-Marroyo, 2013). The combined work of these factors will enable the human body to achieve balance through its neuromuscular system. When the human body is in a bipedal position, it is in an unstable equilibrium, which is continuously restored by its neuromuscular system (García-López and Rodríguez-Marroyo, 2013).

Gallahue distinguishes five stages in human motor development (Gallahue and Ozmun, 2006): phase of reflex movements (0–1 years), phase of rudimentary movements (1–2 years), phase of basic motor skills (2–7 years), phase of specific motor skills (7–11 years), and phase of specialised motor skills (over 11 years). It is at the beginning of the third of the stages cited by this author, basic motor skills, that the development of balance becomes more important through changes of rhythm and turning. Included in the balance skills, balance, together with the development of strength and coordination, will enable the learning and development of other types of more complex movements.

Children of school age are characterised by a motor development in which perceptual-motor abilities begin to have a certain stability. This stability is a consequence of several factors: a greater maturation of the central nervous system and motor learning, the acquisition of the body schema and the acquisition of a greater functional autonomy of the body segments (Ruiz-Pérez et al., 2014). This period of motor stability is due to exogenous factors such as the practice of PA (Gutiérrez, 2008). However, fitness deficits and their most direct consequence, poor physical fitness, are related to motor development problems (Cairney et al., 2005; López-Gallego et al., 2016), among these are low levels of balance. By the age of 11–12 years, all coordination and balance skills should be fully defined and functionally efficient (Ruiz-Pérez et al., 2014). Practising regular PA or following a physical exercise training programme improves balance stability (García-López and Rodríguez-Marroyo, 2013). This improvement is a consequence of increasing the quality of the neuromuscular system (physiological factor) and the person’s fitness levels. In addition, this increase in the quality of the neuromuscular system is associated with an improved emotional state (psychological factor).

One of the factors that has the greatest impact on proper motor development is the regular practice of PA. This regular PA practice is encouraged for the entire world population by the World Health Organisation (WHO), which has published recommendations for PA practice by age group of the population. In the case of children, the WHO recommends at least 60 min of moderate to vigorous intensity PA (MVPA) daily. All the time a child spends in PA above this recommended time will lead to more health benefits (World Health Organization, 2010). It also states that children aged 5–12 should use technology devices and screens for no more than 60–90 min a day (World Health Organization, 2010, 2021), although the data are much higher (Cartanyà-Hueso et al., 2021). Similarly, there is also a preventive effect of regular PA practice with minimal exertional intensity on infectious diseases. Regular PA at least 30 min a day, 5 days a week can reduce the risk of contracting a virus, including COVID-19, by 31%, reduce the probability of death from infectious diseases by 37% and improve the effectiveness of vaccines by up to 40% (Chastin et al., 2021).

These WHO recommendations are far removed from the reality of Spanish children between 9 and 15 years of age, showing very negative data: the highest levels of sedentary lifestyles and abandonment of PA and sport outside the school context, with 85% of girls and 78% of boys. Spanish children in this age group do not spend the minimum number of minutes established by the WHO (World Health Organization, 2021). Also of concern is the population of children who seem to be more in need of regular PA practice, such as overweight or obese schoolchildren as opposed to schoolchildren with a body mass index (BMI) of normal weight (Wickel and Belton, 2016; Cartanyà-Hueso et al., 2021). Both elements, the decrease in the practice of PA and sport and the increase in the time spent using technological devices and screens, constitute the so-called technological sedentary lifestyle (Lozano-Sánchez et al., 2019; Arufe-Giráldez et al., 2020) and may be associated with other health problems (Díaz and Aladro, 2016).

Therefore, the main aim of this research is to determine the implication that lockdown in Spain due to the SARS-CoV-2 virus outbreak had on balance in 11–12 year-old schoolchildren. We investigated the possible relationships established between the balance tests assessed and certain socio-demographic variables (e.g., type of housing, place of residence, length of PA) in the context of school closures.

## Materials and methods

### Study design

In order to carry out this research, a descriptive and longitudinal observational study was conducted (Ato et al., 2013). Children’s balance was the dependent variable used in the study. The independent variables of the research were defined on the basis of an *ad hoc* questionnaire. This questionnaire collected socio-demographic data of the children who participated in the study (e.g., age, gender, parents’ educational level, employment status) as well as data related to the different variables under study (e.g., perception of tiredness, perception of self-esteem, perception of creativity).

### Participants

Fifty-five children from a primary school in northern Spain were invited to take part in the research. These children were in the sixth grade at the time of the research. The school is located in a semi-urban residential area close to the city of Santander. Of the 55 children invited to participate in the study, 50 children finally participated, 33 (66%) boys and 17 (34%) girls (median age = 11.40, SD = 0.50). The other five children were excluded from the study because they did not provide informed consent from their parents or legal guardians or because they chose not to participate. The sample was distributed as follows: 56% resided in an urban setting, 38% in a semi-urban or residential setting and 6% resided in a rural setting during the SARS-CoV-2 closure period.

The participants in this study have contributed to a better understanding of the consequences of home lockdown in Spain due to SARS-CoV-2. In addition to the implications that such lockdown had on their balance, the implications for their manual dexterity are also known (Ramos Álvarez et al., 2022).

### Instruments

The Spanish adaptation of the Movement Assessment Battery for Children 2 (MABC-2) was used for this research (Henderson et al., 2007; Ruiz Pérez and Graupera-Sanz, 2012). The MABC-2 is composed of three dimensions: Dimension 1, which assesses children’s manual dexterity, Dimension 2, which assesses aiming and catching, and Dimension 3, which assesses balance. Each of these dimensions is made up of different tests. The test administered in this research were a static balance test and two dynamic balance test.

The static balance test (B1), balance on two supports, consists of the child standing on the narrow part of some supports and in a heel-toe position for a maximum of 30 s. In order to carry out this test, a wide space must be available where the child cannot lean on it. Specific supports are also required for the execution of this test. The researcher must also demonstrate the correct execution of the test. The investigator must emphasise the importance of a stable position, that it is not permitted to lift the feet off the supports, to touch the base of the supports with the edges of the feet and that they can use their arms to balance themselves. The time shall be counted when the child is perfectly balanced. Before the test is performed, the child has a 15-s trial and has two attempts to achieve the objective of this test.

The first dynamic balance test (B2), walking backward heel-toe, consisted of the child walking backward in a heel-toe pattern along a line made with adhesive tape. The length of this line was 4.5 m. The child has 5 trial steps and two attempts to walk 15 steps or finish the line without mistakes. It is not possible to help the child, but a demonstration should be given, stressing that there should be no gaps between each step, that the child should not step off the line, that he/she should not touch the ground with the free foot to regain balance, and that the position of the foot should not be readjusted once it is on the line.

The last of the tests performed and the second dynamic balance test (B3 and B4 for dominant and non-dominant leg, respectively), is jumping on one leg in a zigzag. To perform this test, specific equipment is required. This material consists of mats: 3 yellow mats, 2 blue mats, and a blue mat with an orange circle (target mat). These mats will be arranged in a zigzag pattern over a distance of 4.5 m. The child must stand on the first mat on one leg and must jump from mat to mat with the same leg to the target mat. The test must be performed on both legs and the child can choose which leg he/she wants to start the test with. As in the other tests, the researcher must demonstrate the limits over which the child must jump on the mats, jumping one mat at a time and without stopping, avoiding the free foot touching the floor or another mat and finishing the jumps in balance and within the target mat. You will have one trial with each leg and two attempts with each leg during your evaluation. The maximum score (five points) is achieved if the child manages to perform all five zigzag jumps without error.

For the performance of the tests, their measurement and subsequent evaluation, the procedures established in the MABC-2 were followed, using specific test materials, as well as a digital hand-held stopwatch and adhesive tape. Likewise, for the interpretation of the research results, the reference values established in the MABC-2 were taken into account (Ruiz Pérez and Graupera-Sanz, 2012).

The data obtained by means of the MABC-2 were complemented by means of an *ad hoc* socio-demographic questionnaire. This questionnaire was completed by the parents or legal guardians and collected information on different variables: economic and educational information on the family, information on the time dedicated to the practice of PA, information on the time dedicated as well as the type of sedentary activities carried out and finally information on the time dedicated and the type of technologies used by the children before and during confinement. The designed questionnaire is composed of 50 dichotomous items, items rated on a Likert scale and open-ended questions. The questionnaire showed an acceptable Cronbach’s alpha coefficient (α = 0,71) (Nunnally and Bernstein, 1994; George and Mallery, 2003).

### Procedure

The research was carried out during the 2019–2020 academic year. Three data collections were conducted: two pre-lockdown data collections and a third post-lockdown data collection. The pre-lockdown data collections were conducted in physical education classes, specifically during the weeks of 14 October 2019 and 2 March 2020. While the post-lockdown data collection took place in the so-called de-escalation period in Spain: during the week of 28 May 2020.

Spain closed its schools indefinitely from 15 March 2020 due to the outbreak of SARS-CoV-2 in Spain (Agencia Estatal Boletín Oficial del Estado, 2020). It is from this date that the national lockdown began in Spain as a result of the state of alarm decreed due to the health emergency caused by SARS-CoV-2. Following this event, all families of the children participating in the research were contacted and informed of the changes that were to be made to the study. These changes affected the post-lockdown data collection process. This new information, conveyed in writing, did not cause any participants to drop out of the study.

In the post-lockdown data collection, the same procedures used in the previous data collection were used and some modifications were introduced to ensure compliance with the health measures established by the Spanish government to prevent the spread of SARS-CoV-2. Firstly, as a consequence of the closure of primary schools, data collection was conducted outside physical education classes. Secondly, groups of six sampled children were convened in different time slots and in an outdoor space for data collection. In addition, in this post-lockdown data collection, data from the socio-demographic questionnaire were collected by means of a paper survey. This questionnaire was completed by the parents or legal guardians. With this third data collection, a statistical analysis was carried out between the results obtained between pre-lockdown 2 and post-lockdown.

### Statistical analysis

The statistical software SPSS v. 26 (IBM Corporation, New York, NY, USA) was used to perform the statistical analyses of the research. Descriptive analyses of the main variables of the research were performed as well as normality tests of the quantitative variables for hypothesis testing. For normality analyses of the total sample, the Kolmogorov-Smirnov statistic was used (*n* > 50). In the case of normality tests by sex, the Shapiro–Wilk statistic was used (*n* < 50).

The Mann–Whitney *U* test for paired samples was also performed to check whether there were statistically significant differences (*p* < 0.05) between the data obtained in the different data collections for the research variables, both for the total sample and by sex. This test was performed on the two pre-lockdown data collections as well as on the post-lockdown data collection. This decision was based on having a sample of less than 25 female participants. The Mann–Whitney *U* test has been shown to be the most appropriate test for investigations with samples similar to the one presented in this study. Numerous scientific articles have addressed the suitability of its use in similar circumstances in the paediatric population (Gómez-Gómez et al., 2003; Ramírez and Polack, 2020; Ortega et al., 2021).

Finally, the Kruskal–Wallis *H*-test was used in the case of three or more groups. This test was applied with the data collected in the family habits questionnaire.

### Ethical aspects

The ethical and deontological principles established by the American Psychological Association have been followed in this research (American Psychological Association, 2020), as well as ethical recommendations for educational research (Paz, 2018).

The research protocol was approved by EDUCA’s Ethics Committee under code 82019.

## Results

### Balance assessment tests and scores

#### Descriptive and functional analysis

To confirm the normality of the assessment scores as well as the balance tests performed, the Shapiro–Wilk test was performed. This test rejects the hypothesis of normality in the two pre-lockdown data collections in the balance dimension score, scalar score and balance percentile in both boys and girls. The results of the Shapiro–Wilk test in the post-lockdown data collection on these same scores also show the rejection of the normality hypothesis for both girls and boys. The exception is the boys’ balance dimension score (*p* = 0.113).

Significant differences were found for the total balance score, the scalar score and the percentile (Table 1).

**TABLE 1:** The balance test results between pre-lockdown 1, pre-lockdown 2, and post-lockdown using descriptive analysis.

	Pre-lockdown 1			Pre-lockdown 2			Post-lockdown		
	*n* (Total)	Boys	Girls	*n* (Total)	Boys	Girls	*n* (Total)	Boys	Girls
SD	30.96 ± 5.41*	31.06 ± 4.98*	30.76 ± 6.32	34.44 ± 2.12	34.15 ± 2.57	35.00 ± 0.00**	26.48 ± 7.11***	25.39 ± 7.18***	28.58 ± 6.67^+^
SDS	10.56 ± 3.15*	10.57 ± 3.06*	10.52 ± 3.41	12.66 ± 1.28	12.48 ± 1.56	13.00 ± 0.00**	7.92 ± 4.12***	7.33 ± 4.10***	9.05 ± 4.03^+^
BP	59.32 ± 29.71*	59.15 ± 30.09*	59.65 ± 29.86	80.46 ± 13.13	78.63 ± 15.93	84.00 ± 0.00**	36.84 ± 32.89***	32.02 ± 31.86***	46.20 ± 33.80^+^
B1	23.36 ± 8.79*	23.78 ± 8.40*	22.52 ± 9.72	29.34 ± 3.42	29.00 ± 4.19	30.00 ± 0.00**	20.80 ± 10.52***	20.36 ± 10.27***	21.64 ± 11.27^+^
B2	13.20 ± 3.73	12.81 ± 3.86	13.94 ± 3.47	14.66 ± 1.23**	14.48 ± 1.50**	15.00 ± 0.00	9.66 ± 4.80***	9.06 ± 4.78***	10.82 ± 4.77^+^
B3	4.98 ± 0.14	5.00 ± 0.00	4.94 ± 0.24	5.00 ± 0.00	5.00 ± 0.00	5.00 ± 0.00	4.96 ± 0.19	4.93 ± 0.24	5.00 ± 0.00
B4	4.94 ± 0.31	4.90 ± 0.38	5.00 ± 0.00	5.00 ± 0.00	5.00 ± 0.00	5.00 ± 0.00	4.74 ± 0.77^+^	4.63 ± 0.92^+^	4.94 ± 0.24

Data are presented as mean ± standard deviation. n = 50; boys = 33; girls = 17. Abbreviation: SD, score dimension balance; SDS, score dimension scaling balance; BP, balance percentile; B1, balance 1—static balance test; B2, balance 2—dynamic balancing test 1; B3, balance 3—dynamic balancing test 2 with dominant leg; B4, balance 4—dynamic balancing test 2 with non-dominant leg. *p < 0.001 = significant for pre-lockdown 2. **p < 0.05 = significant pre-lockdown 1. ***p < 0.001 = significant with pre-lockdown 2. ^+^p < 0.05 = significant with pre-lockdown 2.

In tests for related samples when the *p*-value is significant (*p* < 0.05) the hypothesis is accepted with 95% confidence that there is a statistically significant difference in the mean value of the variable between the different data collections. This circumstance appears in the total sample between the three data collections in the balance dimension score, in the scalar score and the balance percentile (*p* = 0.000). Identical results are found for boys in these scores. In the case of girls, statistically non-significant data appear in the scores between the first pre-lockdown data collection and the post-lockdown data collection: balance dimension score (*p* = 0.230), scalar score (*p* = 0.201), and balance percentile (*p* = 0.114).

In relation to the different tests carried out, between the first and second data collection pre-lockdown, there are statistically significant differences in the balance test on supports. These results are obtained for the total sample, in boys and girls. There are also statistically significant differences in the heel-toe backward walking test in the total sample and in the boys, results that do not occur in the girls (*p* = 0.180). In the rest of the tests performed between the first and second pre-lockdown data collection (zigzag with dominant and non-dominant leg), there are no statistically significant results in the total sample, in the boys or in the girls. Therefore, there was an improvement in the results of the first two balance tests between the two pre-lockdown data collections.

The data analysed above are similar to those obtained between pre-lockdown 2 and post-lockdown. The results show statistically significant differences (*p* < 0.05) in the mean scores of the balance on supports assessment tests and the heel-toe backward walking test. These results are given for the total sample, boys and girls. Statistically significant results are also given in the zigzag test with non-dominant leg in the total sample (*p* = 0.026) and in the boys (*p* = 0.039). In the zigzag test with dominant leg the results are not significant for the total sample (*p* = 0.157), neither for boys (*p* = 0.157) nor for girls (*p* = 1.000).

The research showed a worsening of test scores between pre-lockdown 2 and post-lockdown, except in the zigzag jumping test with dominant leg in girls, which maintained the same score (*p* = 5.00). Likewise, this worsening showed significant differences in the scores of all the balance assessment tests performed, except in the zigzag jump test with dominant leg in the total sample (*p* = 0.157), in the boys (*p* = 0.157) and in the girls (*p* = 1.000), as well as in the jump with non-dominant leg in the girls (*p* = 0.317). The results obtained by gender do not show large differences between boys and girls in the tests performed, but girls obtain better results in the tests performed between pre-lockdown 2 and post-lockdown.

#### Evolution of the tests

The results obtained from the different balance tests performed according to the MABC-2 show improvements between the results of the pre-lockdown 1 and pre-lockdown 2 data collection. This improvement is smaller in the dynamic balance tests of zigzag jumps, which maintained stable results with small modifications between the different data collections.

However, this improvement was not reflected in the post-lockdown results. There was a negative evolution in all the post-lockdown results, even worsening the results obtained in the pre-lockdown data collection 1. This worsening occurs both in the mean values of the sample and by gender, although with less worsening in girls.

**FIGURE 1:**
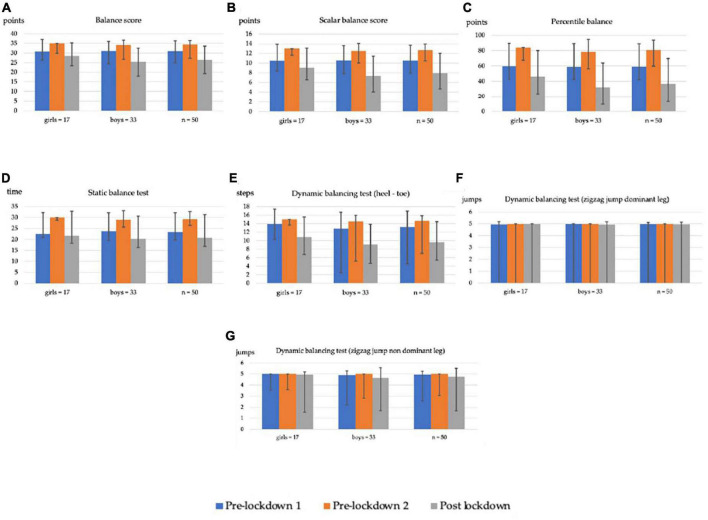
Columns of the evolution of mean balance scores and their different tests with standard deviation bars. **(A)** Balance dimension score; **(B)** Balance scalar score; **(C)** Balance percentile; **(D)** Static balance test–values in seconds; **(E)** Dynamic balance test 1–values in steps performed correctly; **(F)** Dynamic balance test 2 with dominant leg–values in jumps performed correctly; **(G)** Dynamic balance test 2 with non-dominant leg–values in jumps performed correctly.

Table 1 and Figure 1 show the results obtained for Dimension 3 of the MABC-2 in the three data collections carried out in this research (two pre-lockdown and one post-lockdown data collection). These results are broken down by the mean value of the total sample, as well as by gender, in the tests performed as well as in the total, scalar and percentile balance scores.

The MABC-2 (Henderson et al., 2007) establishes three classifications based on the total scores obtained. It classifies in the red zone if the total score is less than 62 points, associated with difficulty in the child’s movement. A second zone is called amber. This Zone is between 63 and 69 points and establishes a certain risk of having movement problems. And finally, a green Zone is established for scores above 69 points and in which children do not present movement problems. According to this classification and the mean scores obtained by the sample in the first data collection, the sample was in the Green Zone: the whole sample (*M* = 71.220), the boys (*M* = 70.636) and the girls (*M* = 72.352).

In the second data collection and even without the sample being confined, the sample experienced a notable improvement in the results obtained in the different tests: 19.23% for the total sample, 19.60% for the boys and 18.53% for the girls. This enabled the sample to remain in the Green Zone: the total sample (*M* = 84.920), the boys (*M* = 84.484) and the girls (*M* = 85.764). The results showed a worsening as a consequence of the period of lockdown due to SARS-CoV-2 in Spain. This aspect was shown in that the means of the boys (*M* = 62.636) and the full sample (*M* = 65.580) both scored within the amber zone. It should be remembered that the amber zone may indicate a risk of movement problems. However, the girls in the sample obtained results that kept them in the green zone, although with worse values than the initial values (*M* = 71.294).

In the specific case of balance and the results obtained from the specific tests set out in the MABC-2, the same worsening occurs. According to the scores obtained in the different tests, specifically the scalar scores, it is observed that the sample starts from normal levels of balance: in the total sample (*M* = 10.56), in the boys (*M* = 10.57) and in the girls (*M* = 10.52). These balance levels undergo a significant improvement in the second pre-lockdown data collection, both in the total sample (*M* = 12.66), in the boys (*M* = 12.48) and in the girls (*M* = 13.00). These scores show no risk of movement in the sample.

However, and as a direct consequence of the lockdown, the balance scores suffer a significant deterioration: in the total sample (*M* = 7.92), in the boys (*M* = 7.33) and in the girls (*M* = 9.05). These scores are not yet within the MABC-2 reference values for movement risk, but the worsening of balance levels in the sample is significant as a consequence of the lockdown.

### Family habits

#### Descriptive analysis

In relation to the data obtained from the questionnaire on family habits during lockdown and completed by the parents or legal guardians of the children, a basic descriptive analysis was carried out. Of note are the data in relation to exposure to screens, educational and cultural activities carried out by the children, hours of rest, the practice of PA or the place and size of residence during lockdown (Ramos Álvarez et al., 2022).

In relation to the place and size of residence during lockdown, this was considered important information for the research as a limiting aspect for the practice of physical activity during confinement. The data show that 6% of the sample resided in a rural setting, 38% in a semi-urban or residential setting, and 56% in an urban setting. In terms of dwelling size, 36% of the sample resided in a house with a garden, 28% in a flat of between 91 and 120 m^2^, 28% in a flat of between 61 and 90 m^2^, and 8% of the sample in a flat of less than 60 m^2^. However, there was no evidence in this study that place of residence and size of dwelling during the period of lockdown had a significant influence on differences in PA minutes or balance scores during lockdown. Two exceptions occurred around place of residence, which showed significant results in the static balance test on supports (*p* = 0.023) and in the dynamic balance test of zigzag jumping with non-dominant leg (*p* = 0.009).

On the practice of physical activity, there was an increase from 4 to 32% of the children in the sample who did not engage in any physical activity. However, during lockdown there was a decrease in the frequency of weekly PA practice. Children who practised PA 2–3 times a week suffered a decrease of 10%, 14% among children who practised between 4 and 5 days and from 10 to 6% among children who had practised between 6 and 7 days. In relation to the amount of rest time children spent during the lockdown period, 20% of the sample slept 8 h a day, 40% slept 9 h, and 38% slept 10 h a day.

In relation to sedentary activities carried out by the sample during lockdown, educational and cultural activities were taken into account in this study, as well as the time devoted by the children to the consumption of screens. Regarding educational and/or cultural activities, 82% of the sample spent more than 60 min doing homework and 30% spent more than 146 min. A total of 38% of the sample spent between 16 and 30 min a day reading, 2% playing musical instruments and 8% spent more than 60 min on artistic activities.

Finally, in relation to the time the children were exposed to screens, the entire sample had a daily exposure to screens of more than 60 min. A total of 52% used a video console, 50% watched TV, 48% used a computer, 30% used a tablet and 26% used a mobile phone.

#### Statistically significant differences

The Mann–Whitney *U*-test and the Kruskal–Wallis *H*-test were used, taking as a reference the recommendations established by the WHO (World Health Organization, 2022) to measure whether there were statistically significant differences between the equilibrium variables and the sociodemographic variables at pre- and post-lockdown. According to the items of interest for this research, these tests of independence showed no statistically significant differences (*p* > 0.05) between the post-lockdown balance variables and the sociodemographic variables, with some exceptions. These exceptions that showed statistically significant differences are: whether any parent is an athlete with respect to the static balance test on supports (*p* = 0.010), the place of residence with respect to the static balance test on supports (*p* = 0.023) and with the dynamic balance test of zigzag jumping with non-dominant leg (*p* = 0.009), the mean number of minutes/day that the child uses a video console with respect to the static balance test on supports (*p* = 0.015) and with the dynamic zigzag jumping balance test with non-dominant leg (*p* = 0.014) and finally the mean number of minutes/day that the child plays a musical instrument with the dynamic zigzag jumping balance test with dominant leg (*p* = 0.000).

#### Post-lockdown results

Once the analyses of the family habits questionnaire and the post-lockdown balance score and test variables had been carried out, a statistical analysis was performed between both types of variables. This analysis focused on the relationship between the results of the post-lockdown balance variables in relation to the frequency of PA practice in the sample.

This analysis shows worsening in three of the four balance tests performed in this study in children who did not practice PA during lockdown: decrease in balance time in the static balance test on supports (−5.68%) and in the dynamic balance tests of zigzag jumps with dominant (−1.4%) and non-dominant (−11.4%) leg. However, there is a maintenance or improvement of the pre-lockdown results after the lockdown period in children who have performed PA. There are three exceptions with notable worsening of results: decrease in the time of balance in the static balance test on supports (−3.59%) in children who performed between 2 and 3 days of PA during lockdown, as well as those who practised between 6 and 7 days a week (−13.91%). Finally, there were also worse post-lockdown results in children who performed 6–7 days of weekly physical activity in the dynamic balance test of zigzag jumping with non-dominant leg (−2.91%). A breakdown of the results can be found in Table 2.

**TABLE 2:** Pre- and post-lockdown balance test scores in relation to the frequency of children who were not physically active using descriptive analysis.

	PA practice			
	No PA	2–3 times/week	4–5 times/week	6–7 times/week
B1 pre-lockdown	21.00 ± 12.72	23.34 ± 9.59	17.05 ± 10.63	24.00 ± 12.32
B1 post-lockdown	19.87 ± 10.26	22.50 ± 11.14	19.61 ± 9.70	20.66 ± 16.16
B2 pre-lockdown	7.00 ± 1.41	10.69 ± 4.71	8.75 ± 4.94	9.60 ± 5.55
B2 post-lockdown	8.43 ± 5.11	11.22 ± 3.84	8.69 ± 5.61	11.00 ± 3.60
B3 pre-lockdown	5.00 ± 0.00	4.95 ± 0.20	4.95 ± 0.22	5.00 ± 0.00
B3 post-lockdown	4.93 ± 0.25	5.00 ± 0.00	4.92 ± 0.27	5.00 ± 0.00
B4 pre-lockdown	5.00 ± 0.00	4.69 ± 0.87	4.75 ± 0.78	4.80 ± 0.44
B4 post-lockdown	4.43 ± 1.20	4.94 ± 0.23	4.84 ± 0.55	4.66 ± 0.57

Data are presented as mean ± standard deviation. PA, physical activity; B1, balance 1—static balance test; B2, balance 2—dynamic balancing test 1; B3, balance 3—dynamic balancing test 2 with dominant leg; B4, balance 4—dynamic balancing test 2 with non-dominant leg.

Another important factor studied in this research is the increased exposure time to screens by the children in the sample. This event may have limited the practice of physical activity during the lockdown period. Table 3 shows the results obtained for the variables analysed by means of the balance tests in relation to the time the sample spent using screens during the confinement period to the detriment of the practice of PA.

**TABLE 3:** The post-lockdown balance test results according to exposure to screens during lockdown using descriptive analysis.

	Average minutes/day of video game console use during lockdown								
	
Test	0	1–15	16–30	46–60	61–75	76–100	101–115	116–130	+146
B1	26.93 ± 7.62	11.00 ± 0.00	30.00 ± 0.00	10.60 ± 10.45	–	21.33 ± 11.86	–	18.33 ± 9.90	16.40 ± 10.78
B2	10.00 ± 5.25	3.00 ± 0.00	9.00 ± 0.00	12.20 ± 4.08	–	12.66 ± 4.08	–	7.00 ± 3.76	12.00 ± 2.82
B3	4.93 ± 0.25	5.00 ± 0.00	5.00 ± 0.00	5.00 ± 0.00	–	5.00 ± 0.00	–	4.93 ± 0.25	5.00 ± 0.00
B4	5.00 ± 0.00	2.00 ± 0.00	5.00 ± 0.00	4.60 ± 0.54	–	5.00 ± 0.00	–	4.46 ± 1.12	5.00 ± 0.00

	**Average minutes/day of television use during lockdown**								
	
**Test**	**0**	**1–15**	**16–30**	**31–45**	**46–60**	**76–100**	**101–115**	**116–130**	**+146**

B1	20.25 ± 11.61	29.00 ± 1.41	10.75 ± 13.09	30.00 ± 0.00	22.00 ± 10.34	20.00 ± 14.14	25.00 ± 0.00	21.76 ± 10.14	15.40 ± 10.80
B2	9.25 ± 4.64	12.00 ± 4.24	5.50 ± 3.10	11.00 ± 5.65	10.38 ± 4.29	10.50 ± 6.36	15.00 ± 0.00	9.88 ± 5.40	7.80 ± 5.49
B3	5.00 ± 0.00	5.00 ± 0.00	5.00 ± 0.00	5.00 ± 0.00	5.00 ± 0.00	5.00 ± 0.00	5.00 ± 0.00	4.94 ± 0.24	4.80 ± 0.44
B4	5.00 ± 0.00	5.00 ± 0.00	4.75 ± 0.50	5.00 ± 0.00	4.53 ± 1.12	5.00 ± 0.00	5.00 ± 0.00	4.70 ± 0.84	4.80 ± 0.44

	**Average minutes/day of PC use during lockdown**								
	
**Test**	**0**	**1–15**	**16–30**	**31–45**	**46–60**	**61–75**	**76–100**	**116–130**	**+146**

B1	21.20 ± 11.35	30.00 ± 0.00	15.80 ± 12.33	30.00 ± 0.00	21.77 ± 9.12	30.00 ± 0.00	22.75 ± 13.20	21.13 ± 11.38	13.75 ± 4.19
B2	10.20 ± 3.96	3.00 ± 0.00	7.40 ± 7.16	7.00 ± 0.00	10.55 ± 4.36	7.00 ± 0.00	12.75 ± 4.50	10.00 ± 5.16	7.75 ± 4.11
B3	5.00 ± 0.00	5.00 ± 0.00	4.80 ± 0.44	5.00 ± 0.00	4.88 ± 0.33	5.00 ± 0.00	5.00 ± 0.00	5.00 ± 0.00	5.00 ± 0.00
B4	4.90 ± 0.31	5.00 ± 0.00	5.00 ± 0.00	5.00 ± 0.00	4.66 ± 1.00	2.00 ± 0.00	4.75 ± 0.50	4.86 ± 0.51	4.25 ± 1.50

	**Average minutes/day of tablet use during lockdown**								
	
**Test**	**0**	**1–15**	**16–30**	**31–45**	**46–60**	**76–100**	**101–115**	**116–130**	**+146**

B1	22.29 ± 10.16	30.00 ± 0.00	17.90 ± 10.93	30.00 ± 0.00	17.80 ± 9.20	19.50 ± 14.84	–	21.55 ± 11.35	17.50 ± 14.52
B2	9.23 ± 4.63	9.00 ± 8.48	9.10 ± 5.46	7.00 ± 0.00	11.60 ± 5.27	10.50 ± 6.36	–	10.66 ± 4.77	8.75 ± 4.78
B3	4.94 ± 0.24	5.00 ± 0.00	4.90 ± 0.31	5.00 ± 0.00	5.00 ± 0.00	5.00 ± 0.00	–	5.00 ± 0.00	5.00 ± 0.00
B4	4.58 ± 1.00	5.00 ± 0.00	4.60 ± 0.96	5.00 ± 0.00	4.60 ± 0.89	5.00 ± 0.00	–	5.00 ± 0.00	5.00 ± 0.00

	**Average minutes/day of mobile phone use during lockdown**								
	
**Test**	**0**	**1–15**	**16–30**	**31–45**	**46–60**	**61–75**	**101–115**	**116–130**	**+146**

B1	20.00 ± 10.49	16.50 ± 19.09	23.33 ± 11.54	28.00 ± 0.00	20.20 ± 10.78	30.00 ± 0.00	–	19.66 ± 12.50	23.33 ± 10.40
B2	9.50 ± 5.24	10.00 ± 7.07	12.00 ± 2.64	12.00 ± 0.00	7.00 ± 2.73	9.00 ± 0.00	–	8.50 ± 5.39	12.16 ± 4.49
B3	4.96 ± 0.19	5.00 ± 0.00	5.00 ± 0.00	5.00 ± 0.00	4.80 ± 0.44	5.00 ± 0.00	–	5.00 ± 0.00	5.00 ± 0.00
B4	4.65 ± 0.89	5.00 ± 0.00	5.00 ± 0.00	5.00 ± 0.00	4.40 ± 1.34	5.00 ± 0.00	–	4.83 ± 0.40	5.00 ± 0.00

Data are presented as mean ± standard deviation. Abbreviation: PA, physical activity; B1, balance 1—static balance test; B2, balance 2—dynamic balancing test 1; B3, balance 3—dynamic balancing test 2 with dominant leg; B4, balance 4— dynamic balancing test 2 with non-dominant leg.

The results show that from 45 min of use of technological devices onward, worse results are produced in the balance assessment tests. It is also evident that the children who used the technological devices between 31 and 45 min compared to those who did not use them, improved the results of the balance tests carried out.

Similarly, and after analysing the time the sample spent on other sedentary activities during the lockdown period (homework, reading, using musical instruments and performing artistic activities), the results showed that the balance values did not worsen in relation to the daily minutes spent on these activities.

## Discussion

The aim of this study was to determine the implication that lockdown in Spain due to the SARS-CoV-2 virus outbreak had on balance in 11–12 year-old schoolchildren.

The results of this study show a negative evolution in the balance scores of a sample of 11- and 12-year-old boys and girls living in a region of northern Spain as a consequence of the SARS-CoV-2 lockdown. This deterioration occurs in the static equilibrium of the sample as well as in the dynamic equilibrium. Similarly, the deterioration in balance values occurs in boys as well as in girls. The research reinforces other studies of the negative impact that lockdown has had on different psychomotor domains in the paediatric age population, such as children’s manual dexterity, and a generalised worsening in global motor coordination (Ramos Álvarez et al., 2022). This worsening is more important for boys than for girls. Research carried out in the province of Tungurahua (Ecuador), showed a slight and significant delay of 10.35% of the sample studied in both the gross and fine motor areas, as well as in coordination, factors of importance in the balance of the paediatric age population (Sánchez-Reyes et al., 2020).

Similar studies have shown the deterioration in children’s motor skills, including stability and balance, during periods of restricted movement such as the COVID-19 lockdown (Pombo et al., 2021). In this research it was found that motor competence in Portuguese children worsened significantly overall as well as in individual tests after restraint. On this occasion the worsening was not significantly greater in boys than in girls, but worsened equally for both.

Events such as home lockdown are detrimental situations for children’s physical activity behaviours that affect all spheres of human motor skills (Wang G. et al., 2020). As a consequence of this lockdown and the decrease in children’s physical activity time, levels of overweight and obesity have also risen. These pathologies have become a global concern because of the emergence of associated diseases in adulthood (Wang H. et al., 2020). But also because overweight and obese children have poorer levels of balance and motor coordination, which has been accentuated during the COVID-19 lockdown period (Lizondo-Valencia et al., 2021).

Other research has shown that sedentary lifestyles have an important impact on some factors associated with balance. Low rates of regular PA in children and adolescents have negative repercussions on their mental and physical health, and lead to alterations in the musculoskeletal system, postural imbalances and the appearance of back, head, and neck pain. Psychological factors are also affected. These events are more prevalent in girls, who present a higher rate of clinical manifestations of pain related to sedentary lifestyles (Martínez-López et al., 2015). For this reason, strength training is particularly important for improving coordination and balance in children. In addition to preventing possible injuries during the practice of physical sports activities (Kordi et al., 2016; Comité Nacional de Medicina del Deporte Infantojuvenil, 2018).

In relation to this type of study, in Spain, children and adolescents do not meet the physical activity recommendations established by the WHO as optimal for an active and healthy life (Ventura et al., 2021; World Health Organization, 2022). These data have suffered a decrease due to home lockdown due to the outbreak of SARS-CoV-2, with the main consequences being the worsening of physical fitness values as well as anthropometric values in children and adolescents (Maltagliati et al., 2021; Ramos Álvarez et al., 2021a,b; Ventura et al., 2021). Balance has as a group of influencing factors in its development the levels of physical fitness (García-López and Rodríguez-Marroyo, 2013). Therefore, the decrease in the values of equilibrium evidenced in this research may have as one of its causes the loss of physical condition of the sample.

It should be noted that in this pandemic context, several studies have found (Chen et al., 2020; Ortiz and Villamil, 2020; Chastin et al., 2021) that good physical fitness and regular physical activity are preventive factors against SARS-CoV-2 infection. Likewise, optimal physical fitness resulting from regular PA reduces the suffering from the disease. This has the consequence that in case of infection with the virus, it may have lower health risks. This evidence is supported by some of the direct consequences that regular PA practice has on the body: anti-inflammatory, anti-fibrotic and antioxidant effects, which can mitigate the negative effects that COVID-19 can have on the body.

This research has also shown changes in certain habits of the sample that have a direct impact on physical fitness (Ramos Álvarez et al., 2021a), including their performance in balance tests. A change has occurred in the sample’s rest and hours of sleep. For the age range of the sample in this study, the WHO recommends more than 11 h of rest per day, which the sample did not comply with (Ramos Álvarez et al., 2021a).

This research has also evidenced changes in habits between pre- and post-lockdown and that these changes influenced their equilibrium values: related to food and PA. These changes have been evidenced in different studies and with direct implications in different population settings (Arufe-Giráldez et al., 2020; Villaseñor et al., 2020; Santos-Miranda et al., 2021). Another habit that should be highlighted due to the significant changes in all age groups is related to the consumption of time spent with technological devices. There has been a significant increase in the amount of time spent on technology and screen time. This change in habits has a significant impact on the increase in time spent on sedentary activities and a significant loss of time and frequency spent on physical sports activities. This loss of time and frequency devoted to PA leaves values below the WHO’s global recommendations (World Health Organization, 2021). These sedentary habits have become a concern for some States, issuing laws limiting the use of technology by children and adolescents to This, compounded by the SARS-CoV-2 health crisis, has led some governments to implement measures to limit the use of technology, prevent addiction and mitigate the high levels of sedentary behaviour among children and adolescents (Rtve, 2021).

This research, like any other similar study, has certain limitations. The main limitation of the research is the size of the sample and the difference between genders. This limitation prevents the results obtained in this research from being generalisable to the entire Spanish population aged 11–12 years.

Despite the limitations of this study, the research cited in this article shows profiles similar to the characteristics of the sample presented in this research. In addition to the research cited, other documents consulted (especially governmental) corroborate this assertion. The latest survey of the National Statistics Institute (INE) of 2020 (Instituto Nacional de Estadística, 2020) and EUROSTAT reports (Oecd/European Observatory on Health Systems and Policies, 2019) are some examples of government documents that show similar profiles to the sample presented in this research.

## Conclusion

The main objective of this research was to determine the implication that lockdown in Spain due to the SARS-CoV-2 virus outbreak had on balance in 11–12 year-old schoolchildren. The results have shown that there has been an impact on balance as a consequence of this lockdown. This worsening in the equilibrium values of the sample studied may have a multifactorial origin (Ruiz Pérez and Graupera-Sanz, 2012). The same conclusion has been reached in similar studies (Ramos Álvarez et al., 2021a,^2022^). These factors are based on the decrease in time dedicated to the practice of PA, the increase in the use of electronic devices and the increase in daily time dedicated to other types of sedentary activities. This worsening of post-lockdown balance values is lower in girls than in boys. Despite this decrease in the balance values of the total sample as well as by gender, it does not imply that the sample is in balance-related movement difficulties according to the reference values of the MABC-2.

The research has two main limitations that prevent the results obtained in this study from being generalisable to the rest of the population of this age group. The first limitation is the size of the sample, as a larger sample would be needed to be able to generalise the results obtained. The second limitation, also related to the sample, is the difference in sample size between boys and girls. It would have been more desirable for the study to have a more gender-equitable sample. However, these limitations in relation to the sample do not prevent us from highlighting the deterioration that the sample has undergone in its static and dynamic equilibrium as a consequence of the SARS-CoV-2 confinement in Spain. This research is a complement to the research being carried out in different areas of knowledge on the impact of COVID-19 confinement on the paediatric population.

This research reinforces the findings of other research related to the impact of SARS-CoV-2 lockdown in this age group (Ramos Álvarez et al., 2021a,^2022^): the need to reinforce and work on more effective strategies to promote the practice of healthy PA on a regular basis among Spanish children and adolescents is evident. To achieve these objectives, the promotion and development of effective strategies for adherence to the practice of PA among the children participating in this research should be addressed.

Likewise, the work proposed for the promotion of regular PA practice in children and adolescents must be complemented with other educational actions. These educational actions should be linked to the learning and use of effective strategies for the responsible use of new technologies, especially in exceptional situations such as this large-scale home lockdown due to SARS-CoV-2. However, these strategies will not only be useful for exceptional moments such as lockdown, but should also help them to lead a more active life from a motor point of view. This work must be approached in a collaborative way between the different social agents involved in the education of children and adolescents, such as the family, educational centres and local sports organisations.

The problems of Spanish children and adolescents, with low levels of PA, high rates of childhood overweight and obesity, and the worrying consequences of the great lockdown in Spain, seem to be powerful arguments to focus efforts on working on these strategies and solutions.

## Data availability statement

The raw data supporting the conclusions of this article will be made available by the authors upon request. Requests to access the datasets should be directed to OR-Á, oliver.ramos@unican.es.

## Ethics statement

The research protocol was approved by EDUCA’s Ethics Committee under code 82019. Written informed consent to participate in this study was provided by the participants’ legal guardian/next of kin.

## Author contributions

OR-Á, VA-G, AS-R, and RN-P: conceptualisation, validation, writing—review and editing, visualisation, and supervision. OR-Á and VA-G: methodology. OR-Á: software, formal analysis, investigation, resources, data curation, writing—original draft preparation, and project administration. All authors have read and agreed to the published version of the manuscript.
